# Risk of bladder cancer in patients with diabetes mellitus: an updated meta-analysis of 36 observational studies

**DOI:** 10.1186/1471-2407-13-310

**Published:** 2013-06-26

**Authors:** Zhaowei Zhu, Xianjin Wang, Zhoujun Shen, Yingli Lu, Shan Zhong, Chen Xu

**Affiliations:** 1 Department of Urology, Ruijin Hospital, School of Medicine, Shanghai Jiaotong University, Shanghai, China; 2 Institute and Department of Endocrinology and Metabolism, Shanghai Ninth People's Hospital, School of Medicine, Shanghai Jiaotong University, Shanghai, China; 3 Department of Embryology and Histology, School of Medicine, Shanghai Jiaotong University, Shanghai, China; 4 Shanghai Key Laboratory of Reproductive Medicine, School of Medicine, Shanghai Jiaotong University, Shanghai, China

**Keywords:** Diabetes, Bladder Cancer, Meta-analysis

## Abstract

**Background:**

Increasing evidence suggests that a history of diabetes mellitus (DM) may be associated with an increased risk of bladder cancer. We performed a systematic review with meta-analysis to explore this relationship.

**Methods:**

We identified studies by a literature search of Medline (from 1 January 1966) and EMBASE (from 1 January 1974), through 29 February 2012, and by searching the reference lists of pertinent articles. Summary relative risks (RRs) with corresponding 95% confidence intervals (CIs) were calculated with a random-effects model.

**Results:**

A total of 36 studies (9 case–control studies, 19 cohort studies and 8 cohort studies of patients with diabetes) fulfilled the inclusion criteria. Analysis of all studies showed that DM was associated with an increased risk of bladder cancer (the summary RR = 1.35, 95% CI 1.17–1.56, p < 0.001, I^2^ = 94.7%). In analysis stratified by study design, diabetes was positively associated with risk of bladder cancer in case–control studies (RR = 1.45, 95% CI 1.13-1.86, p = 0.005, I^2^ = 63.8%) and cohort studies (RR = 1.35, 95% CI 1.12-1.62, p < 0.001, I^2^ = 94.3%), but not in cohort studies of diabetic patients (RR = 1.25, 95% CI 0.86–1.81, p < 0.001, I^2^ = 97.4%). The RRs of bladder cancer were 1.38 (1.08-1.78) for men and 1.38 (0.90-2.10) for women with diabetes, respectively. Noteworthy, the relative risk of bladder cancer was negatively correlated with the duration of DM, with the higher risk of bladder cancer found among patients diagnosed within less than 5 years.

**Conclusions:**

These findings support the hypothesis that men with diabetes have a modestly increased risk of bladder cancer, while women with diabetes were not the case.

## Background

Bladder cancer represents the first and second most common genitourinary malignancy in China and the USA, respectively. Based on incidence and mortality data from several agencies, the American Cancer Society estimates that 73,510 new bladder cancer cases and 14,880 deaths from bladder cancer are projected to occur in the United States in 2012 [1]. To explore the effective tools for prevention of bladder cancer, great investment has been made to gain new insight into how environmental and genetic factors influence the development of bladder cancer. To date, several risk factors, such as paint, smoking and human papillomavirus infection, have been implicated in urinary bladder carcinogenesis [2-4].

Diabetes mellitus (DM) is considered to be one of the major public health challenges in both industrialized and developing countries [5]. The relationship between DM and malignancies has been investigated extensively; and ample evidence indicates that individuals with diabetes have increased risk of several malignancies, including cancers of the colon and rectum [6]. A clarification of the association between DM and cancer is important for disease prevention and management.

Diabetes may also be a risk factor for bladder cancer, but findings from epidemiological studies are inconsistent. A previous meta-analysis of 16 studies (7 case–control studies, 3 cohort studies and 6 cohort studies of patients with diabetes) conducted in 2006 showed that diabetes was associated with an increased risk of bladder cancer in case–control studies and cohort studies, but not in cohort studies of patients with diabetes. However, a publication bias against studies with small sample sizes and against reporting a low relative risk is possible, and may have resulted in an overestimation of the relationship between diabetes and bladder cancer [7]. Besides, most studies included in the meta-analysis were performed in Western countries, and only one study was conducted in the Asian population in Korea [8]. Thus, the association between DM and bladder cancer in Asian population has not been investigated extensively. Moreover, the association in different gender groups is worthy of investigation, but has not been looked at.

Since the meta-analysis was published, a variety of relevant studies on this association have also yielded inconsistent results. No association was found between DM and bladder cancer in a prospective study of Swedish men [9]. However, a case–control study in New England showed that history of diabetes was related to an increased bladder cancer risk, and the association was strongest in those who had diabetes for the longest duration [10]. Currently, we aim to analyze this relationship further by conducting an updated meta-analysis of relevant studies. This updated analysis of 36 studies will allow us to provide more precise risk estimates than the previous analysis. Furthermore, we also examined whether the association between a history of DM and the risk of bladder cancer differs according to various study characteristics.

## Methods

### Search strategy

A computerized literature search was performed in Medline (from 1 January 1966) and EMBASE (from 1 January 1974), through 29 February 2012, by two independent investigators. We searched the relevant studies with the following text words and/or Medical Subject Headings: ‘diabetes mellitus’, ‘diabetes’, ‘bladder cancer’, ‘urinary bladder neoplasms’, ‘transitional cell carcinoma of the bladder’, and ‘epidemiologic studies’. The search was restricted to articles published in English and reporting on the association between diabetes and bladder cancer in humans. References of relevant review articles and trials were screened for relevant articles that were not found through the database searches. Our systematic review was conducted according to the meta-analysis of observational studies in epidemiology guidelines.

### Selection criteria

In this meta-analysis, we included studies that fulfilled the following criteria: (1) presented original data from case-control or cohort studies; (2) one of the exposure of interest was DM; (3) one of the outcome of interest was bladder cancer; and (4) reported relative risk (RR), odds ratio, or standardized incidence/mortality rate (SIR/SMR) with their 95% confidence intervals (CIs), or provided sufficient information to calculate them. In the event of multiple publications from the same study population, the most recent publication with the largest number of bladder cancer cases was included in the meta-analysis. We did not consider studies in which the exposure of interest was type 1 diabetes, which was defined as early age (≤ 30 years) of diagnosis. Articles or reports from non-peer-reviewed sources were not included in our analysis.

### Data extraction

Two investigators independently assessed and extracted the data into a standardized data extraction form from each publication. Disagreements were resolved by a third author. We did not contact authors of the original studies in the case of missing data. Relevant data included the first author’s last name, publication year, year of the study conducted, study design, study location, source population, sample size (cases and controls or cohort size), measure of exposure and outcome, length of follow-up (if applicable), variables adjusted in the analysis, and the risk estimates with corresponding 95% CIs. If studies reported both incidence and mortality rate, we extracted the incidence rate, as mortality rate could be confounded by survival-related factors. From each study, we extracted the RR estimate that was adjusted for the greatest number of potential confounders.

### Statistical analysis

We included in this meta-analysis studies reporting different measures of relative risks: rate ratio, hazard ratio and SIR/SMR. In practice, these measures of effect yield similar estimates of RR because the absolute risk of bladder cancer is low. The variance of the log RR from each study was calculated by converting the 95% CI to its natural logarithm by taking the width of the CI and dividing by 3.92. Summary relative risk estimates with corresponding 95% CIs were derived using the method of DerSimonian and Laird with the assumptions of a random-effects model, which considers both within-study and between-study variations. When sex-specific estimates were available, we first analyzed together (as RR estimates for bladder cancer) and then separately (as RR estimates for cancers of different gender group).

In assessing heterogeneity among studies, we used the Cochran Q test and I^2^ statistics. These were used to test whether the differences obtained between studies were due to chance. For the Q statistic, a p value of less than 0.10 was used as an indication of the presence of heterogeneity; for I^2^, a value >50% was considered a measure of severe heterogeneity. To explore the potential heterogeneity between studies, we conducted analyses stratified by study design, gender, geographic region, publication year, and we also evaluated the impact of adjustment for age, sex, smoking, alcohol consumption, body mass index (BMI),physical activity on the association between diabetes and the risk of bladder cancer. Studies which reported separate RRs for mutually exclusive categories of duration since diabetes was diagnosed (e.g. < 5 years, ≥5 years) were pooled separately to examine how the strength of the association varied with duration of diabetes.

Publication bias was evaluated using a funnel plot of a trial’s effect size against the SE. Because funnel plots have several limitations and represent only an informal approach to detect publication bias, we further carried out formal testing using the test proposed by the Begg’s adjusted rank correlation test and by the Egger’s regression test [11,12]. All statistical analyses were performed using STATA, version 11.0 (STATA, College Station, TX, USA). A two-tailed p-value of less than 0.05 was considered to be statistically significant.

## Results

### Search results

**Figure 1:**
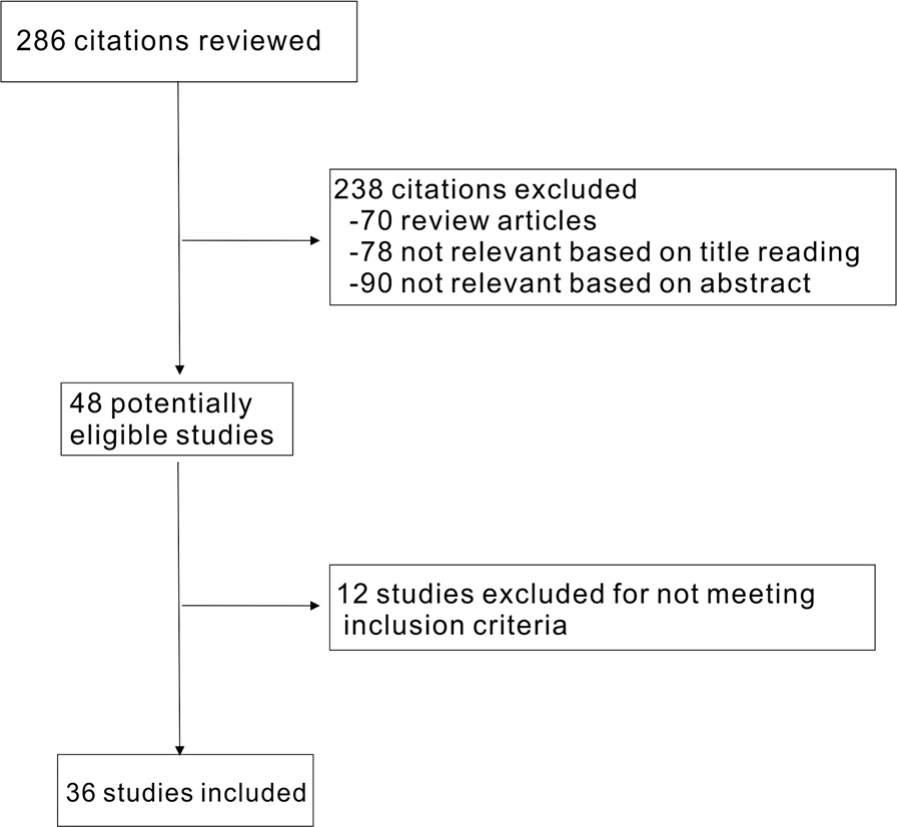
Flow chart on the articles selection process.

We identified 286 potentially relevant articles (Figure 1). After exclusion of duplicate references, none-relevant literature, and those that did not satisfy inclusion criteria, 36 candidate articles were considered for the meta-analysis, including nine case–control studies (Additional file 1: Table S1) [10,13-20], 19 cohort studies (Additional file 2: Table S2) [8,9,21-37] and eight cohort studies (Additional file 3: Table S3) [38-45] of patients with diabetes using external population comparisons. Of these studies, 12 studies were conducted in Europe, 13 in North America, nine in Asia, and two in multiple countries. The study population in 31 studies consisted of men and women, four studies consisted entirely of men and one study included women only. Among these 36 studies, 18 studies did not demonstrate a significantly increased risk of bladder cancer in patients with DM, and the rest studies reported a significantly increased risk of bladder cancer in individuals with diabetes. Potential confounders were controlled in most of the studies, except in nine studies where the adjusted confounders were not clearly indicated.

### DM and risk of bladder cancer

**Figure 2:**
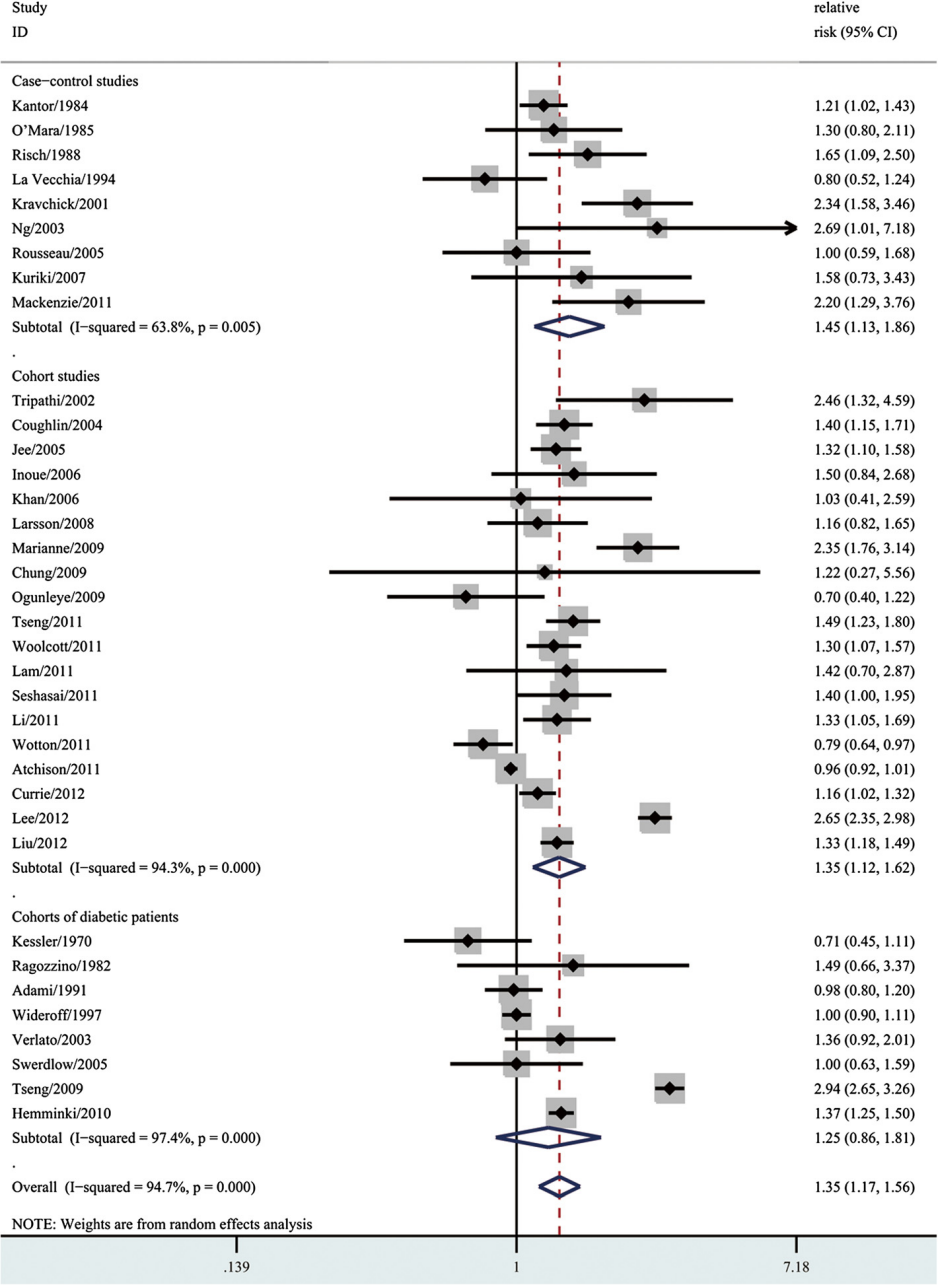
Forest plots of bladder cancer incidence/standard incidence rate associated with diabetes.

As shown in Figure 2, the summary RR with 95% CI was 1.35 (95% CI, 1.17–1.56) in a random-effects model for patients with diabetes, compared with individuals without DM. There was statistically significant heterogeneity among these studies (Q = 660.30, P < 0.001, I^2^ = 94.7%). In analysis stratified by study design, the summary RR with 95% CI was 1.45 (95% CI, 1.13-1.86) and 1.35 (95% CI, 1.35-1.62) in case–control and cohort studies, respectively. However, diabetes was not associated with risk of bladder cancer in cohort studies of patients with diabetes (RR = 1.25; 95% CI, 0.86–1.81). There was statistically significant heterogeneity among the case–control studies (Q = 22.13, p = 0.005, I^2^ = 63.8%), the cohort studies (Q = 315.87, p < 0.001, I^2^ = 94.3%) and the studies of patients with diabetes (Q = 264.47, p < 0.001, I^2^ = 97.4%).

We also investigated the impact of confounding factors on the estimates of relative risk (Additional file 4: Table S4). The summary estimates were significantly higher for studies conducted in Asia and North America (p < 0.05) than in Europe and for studies published in 2000 or later than for studies published before 2000 (p < 0.05). The summary estimates were lower for studies that reported age-adjusted RRs than for those which did not [summary RR (95% CI); 1.34 (1.18-1.51) versus 1.60 (1.53-1.67)]. The summary estimates were higher for studies that reported smoking-adjusted RRs than for those which did not [summary RR (95% CI); 1.32 (1.24–1.39) versus 1.26 (0.99–1.60)]. There was statistically significant heterogeneity within most subgroups.

### DM and incidence of bladder cancer by sex

**Figure 3:**
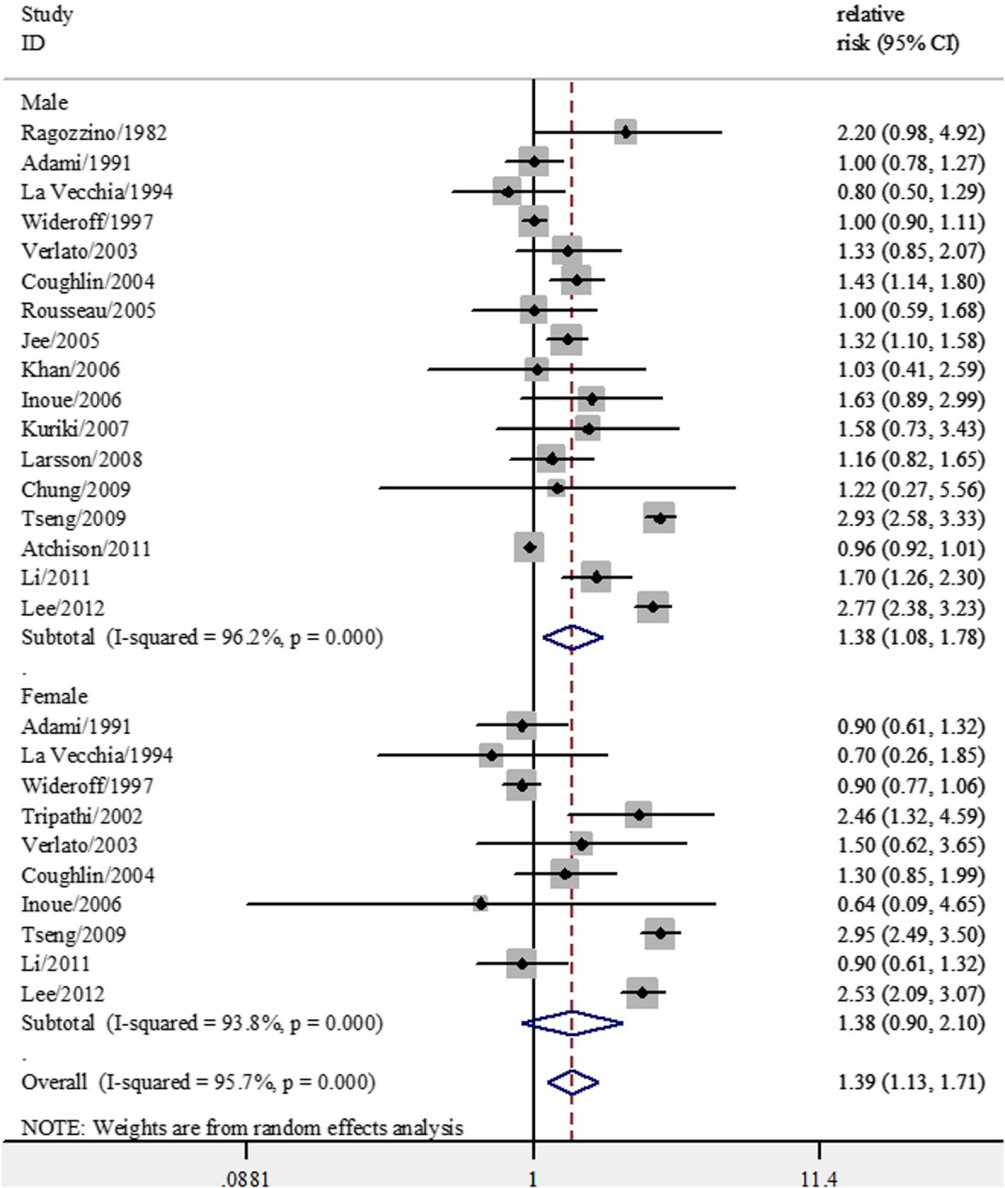
Forest plots of bladder cancer incidence/standard incidence rate by sex associated with diabetes.

Fourteen studies provided results on cancer incidence or mortality specific for gender; Additional four studies consisted entirely of men and one study consisted entirely of women. In stratified analyses by gender, a significantly stronger positive association was observed in men (summary RRs, 1.38; 95% CI, 1.08-1.78; p < 0.001 for heterogeneity). However, diabetes was not associated with an increased risk of bladder cancer in women (summary RRs, 1.38; 95% CI, 0.90-2.10; p < 0.001 for heterogeneity) (Figure 3).

### Duration of DM and risk of bladder cancer

The temporal sequence between diabetes and bladder cancer has not always been clear. Three studies in our meta-analysis presented with RRs for duration of diabetes [10,24,33]. However, duration of diabetes was not similar across the studies. Thus, we categorized the patients into two groups, those with diabetes of less than 5 years and those with diabetes of 5 years or more. Combining these studies according to diabetes duration, we found that individuals with the shorter duration of diabetes (< 5 years) had higher risk of developing bladder cancer than individuals who had duration of diabetes more than 5 years [summary RR, 95% CI; 1.52 (1.05–2.21) versus 1.08 (0.91–1.28)].

### Publication bias

**Figure 4:**
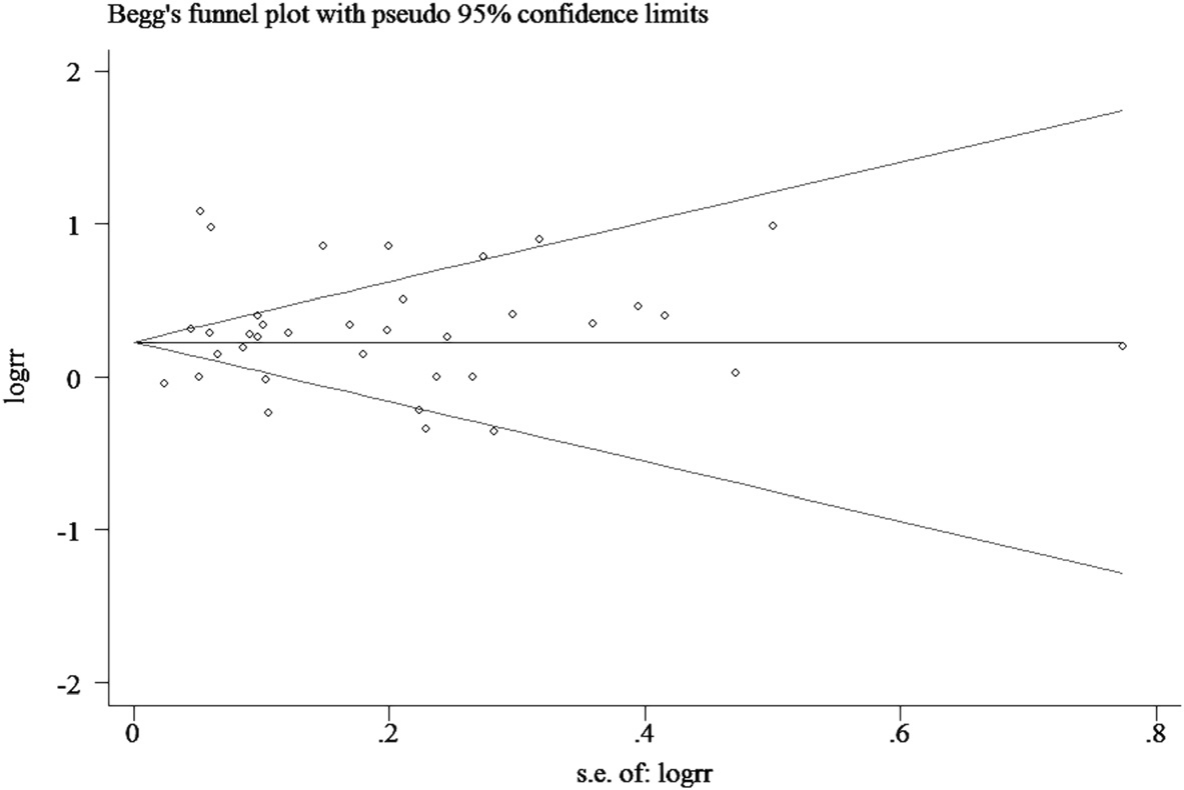
Funnel plot of observational studies evaluating the association between diabetes and bladder cancer risk.

There was no funnel plot asymmetry for the association between DM and risk of bladder cancer. P values for Begg’s adjusted rank correlation test was 0.989 and the Egger’s regression asymmetry test was 0.284, suggesting a low probability of publication bias (Figure 4).

## Discussion

In this meta-analysis, we found that compared with non-diabetics or general population, individuals with diabetes may have more than 35% increased risk of bladder cancer. However, there were differences in the summary RR among different study designs. Whereas diabetes was positively associated with an increased risk of bladder cancer in case–control and cohort studies, the summary estimate in cohort studies of patients with diabetes did not indicate an excess risk of bladder cancer in these cases compared with the general population.

The strength of the study includes that, on an international scale, there are far more individuals with diabetes in our study. After adjustment for important covariates, our study extends previous meta-analysis by providing a more precise estimate of the association between diabetes and bladder cancer risk (based on 36 studies). Despite similar summary RRs between women and men, the positive association was only observed in men, and was independent of BMI, alcohol consumption, smoking status and physical activity. Lorente and colleagues found that transitional cell carcinoma of the bladder was more frequent in males than females. However, never-smoker women have larger and more aggressive tumors with a higher frequency of muscle-invasive disease than male never-smokers and equaling to male current-smokers [46]. Moreover, women also had higher risk of invasive bladder cancer than men even they smoked comparable amount of cigarettes as men [47]. Further studies are needed to confirm our findings and to understand the molecular pathways that might explain the gender-related differences.

Furthermore, the relationship between duration of exposure to diabetes and risk of bladder cancer could be calculated by summing up data from different studies. The current meta-analysis indicated that the risk of bladder cancer was inversely associated with the duration of DM. There was a slightly increased risk which did not reach statistical significance among individuals with diabetes more than 5 years and an increased rate of bladder cancer was observed in individuals with a shorter duration of diabetes (< 5 years). This finding indicated that those with newly diagnosed diabetes should be highly alert to bladder cancer development. However, MacKenzie and colleagues found that compared with those without diabetes, the risk of bladder cancer was highest among those with diabetes of 16 years or more [10]. It is worth noticing that only three studies in our study presented with RRs for duration of diabetes [10,24,33]. When the effect of diabetes was evaluated, glucose-lowering therapies should be adjusted for, and this was not done in most studies. With increasing diabetes duration, the impact of anti-diabetic drugs may set in and influence the association. Thus, the long-term risk of bladder cancer among patients with diabetes warrants further investigation.

In stratified analysis by geographic regions and publication year, we found that the association between DM and bladder cancer was not significant for studies conducted in Europe and for studies published from 1970–1999, whereas studies from North American and Asia and studies published since 2000 showed significantly stronger risk estimates. The regional and temporal differences are perplexing. Many environmental and personal determinants are related, including : genetic factors, lifestyle (eating habits, physical activities, somatotype characteristics), environmental factors (environmental pollution, stress, socioeconomic status ), public health services and so on. With the gradual improvement of medical conditions, early screening and diagnosis rates of DM and bladder cancer are greatly improved. These factors are all attributable to the regional and temporal differences. Our study also has several potential limitations of the available data. Thus, caution is needed when interpreting these results. First, great heterogeneity existed in terms of geographical region, study design, publication year, gender, duration of diabetes and adjustment for confounders. Despite the use of appropriate meta-analytic techniques with random-effect models, we could not account for these differences. The heterogeneity of risk estimates may be due to different mixtures of type 1 and type 2 participants with diabetes and different adjustment for potential confounders. Moreover, some studies included both sexes, whereas others included only men or only women. Nevertheless, subgroup analyses showed that the risk estimate was robust across various quality components.

Second, because diabetes is an underdiagnosed disease, some misclassification of exposure is likely, which would tend to attenuate any true association between diabetes and bladder cancer. In cohort studies of patients with diabetes, the negative association between diabetes and bladder cancer may be due to that the comparison group includes individuals with diabetes, resulting in underestimation of the true effect size.

Third, recent studies have suggested that use of pioglitazone (a common anti-diabetic drug) was associated with an increased incidence of bladder cancer [48,49]. However, most studies included in this meta-analysis did not adjust for the effect of anti-diabetic drugs, which may distort the true relationship between diabetes and risk of bladder cancer.

Forth, confounding cannot be fully excluded as a potential explanation for the observed association, because our analyses were based on observational studies. It is generally accepted that diabetes and bladder cancer share several common risk factors. Smoking has consistently been associated with increased risk of diabetes and bladder cancer [50,51]. The relationship between diabetes and bladder cancer was stronger and statistically significant when we restricted the analysis to those studies which controlled for smoking. When risk estimates from the five studies that adjusted for physical activity were combined, the association between diabetes and bladder cancer was also stronger (RR 1.43) than the overall result including all studies (RR 1.35). In the current analysis, however, adjustment for a wide range of potential confounders, including sex, BMI and alcohol consumption, did not significantly alter the relationship between diabetes and risk of bladder cancer.

Fifth, different study designs may have particular methodological issues and constraints; yet, a common theme with all is the potential for bias. Case–control studies are susceptible to recall and selection biases which could inflate the RRs. Cohort studies are prone to be influenced by detection bias because patients with diabetes are under increased medical surveillance. If medical surveillance bias is present, bladder cancer would tend to be diagnosed at an earlier stage in patients with diabetes than in those without diabetes.

Finally, inherent in any meta-analysis of published data is the possibility of publication bias, that is small studies with null results tend not to be published. However, the results obtained from funnel plot analysis and formal statistical tests did not provide evidence for such bias.

Although the absolute risks of bladder cancer are low among individuals with diabetes, our results have important clinical and public health significance. As a serious and growing health problem in USA, DM affects nearly 25.8 million people, 8.3% of the U.S. population in 2010. In China, a cross-sectional study from 2007 through 2008 involving a nationally representative sample of 46,239 adults, the age-standardized prevalences of total diabetes and prediabetes were 9.7 and 15.5%, respectively [52]. Due to growing obesity epidemic, the prevalence of diabetes will probably increase and contribute to the development of bladder cancer.

## Conclusion

In conclusion, the results from this meta-analysis support an association between diabetes and increased risk of bladder cancer. Further analysis indicates that the positive association is only in men, but not in women. More researches, both epidemiological and mechanistic, are needed to further clarify the association between diabetes and risk of bladder cancer. Future work should also focus on identifying the potential mechanisms underlying this positive link.

## Abbreviations

DM: Diabetes mellitus; CI: Confidence interval; RR: Relative risk; SIR: Standardized incidence rate; SMR: Standardized mortality rate; MI: Body mass index.

## Competing interests

All authors declare that they have no competing interests.

## Authors’ contributions

ZS and YL conceived the study. Data was acquired independently by SZ and CX. ZZ undertook data analysis and interpretation. ZZ and WX prepared the manuscript with contributions from all co-authors. All authors read and approved the final manuscript.

## Pre-publication history

The pre-publication history for this paper can be accessed here:

http://www.biomedcentral.com/1471-2407/13/310/prepub

## Supplementary Material

Additional file 1: Table S1 Characteristics of nine case-control studies of diabetes and bladder cancer risk. Click here for file

Additional file 2: Table S2 Characteristics of 19 cohort studies of diabetes and bladder cancer risk based on rate ratio and hazard ratio. Click here for file

Additional file 3: Table S3 Characteristics of eight cohort studies of diabetes and bladder cancer based on standardized incidence/mortality ratio. Click here for file

Additional file 4: Table S4 Subgroup analysis of relative risks for the association between diabetes and bladder cancer risk. Click here for file
