# Supplementary material for: Risk of bladder cancer in patients with diabetes mellitus: an updated meta-analysis of 36 observational studies
Source: BMC Cancer. 2013 Jun 26;13:310. doi: 10.1186/1471-2407-13-310 (PMC3699355; doi:10.1186/1471-2407-13-310)
Supplement: Additional file 1: Table S1 — Characteristics of nine case-control studies of diabetes and bladder cancer risk. [file 1471-2407-13-310-S1.doc]

**Table 1 Characteristics of nine case-control studies of diabetes and bladder cancer risk**

|  | **No. of**  **subjects** | **Demographics of all subjects (age, years;** **gender, %)** | **Diabetes assessment** | **Bladder cancer assessment** | **Adjusted RR (95% CI)** | **Adjustments** |
| --- | --- | --- | --- | --- | --- | --- |
| Kantor et al. (USA) | 8,764 | Age: 21-84; m: NA | Self-reported | Cancer registries | 1.21 (1.00-1.40) | Sex, race, age, smoking |
| O’Mara et al.(USA) | 19,748 | Age: 30-89; m: 53.6% | Self-reported | Medical records | 1.3 (0.8-2.1) | Age |
| Risch et al. (Canada) | 1,618 | Age: 35-79; m: NA | Self-reported | Medical records | 1.65 (1.09-2.50) | Smoking |
| La Vecchia et al. (Italy) | 17,825 | Age: ＜75; m: NA | Self-reported | Medical records | 0.8 (0.5-1.3) (m)  0.7 (0.3-2.1) (f)  0.8 (0.5-1.2) (both) | Age, sex |
| Kravchick et al.  (Israel) | 801 | Age: 72; m: 80% | NA | Medical records | 2.34 (1.6-3.5) | Age, sex, smoking, DM |
| Ng et al. (UK) | 205 | Age:＞60; m: NA | Medical records | Medical records | 2.69 (1.01-7.19) | Age, smoking |
| Rousseau et al. (Canada) | 3,616 | Age: 59; m:100% | Self-reported | Medical records | 1.0 (0.6-1.7) | Age, BMI, smoking, income, years of schooling, ethnicity, proxy status, β-carotene, coffee, exposure to aromatic amines |
| Kuriki et al. (Japan) | 59,440 | Age: 59; m: 33% | Self-reported | Cancer registries | 1.58 (0.73–3.44) (m) | Age, BMI, alcohol, smoking, physical activity, bowel movement, family history of cancer or DM, dietary restriction, raw vegetable intake, greasy foods intake and snacking |
| Mackenzie et al. (USA) | 594 | Age: 61; m: 68% | Self-reported | Cancer registries | 2.2 (1.3-3.8) | Age, gender, smoking, BMI, and UTI |

Abbreviations: *RR* relative risk, *CI* confidence interval, *DM* diabetes mellitus, *m* male, *f* female, *BMI* body mass index, *NA* data not available, *UTI* urinary tract infeciton
