# Supplementary material for: Risk of bladder cancer in patients with diabetes mellitus: an updated meta-analysis of 36 observational studies
Source: BMC Cancer. 2013 Jun 26;13:310. doi: 10.1186/1471-2407-13-310 (PMC3699355; doi:10.1186/1471-2407-13-310)
Supplement: Additional file 2: Table S2 — Characteristics of 19 cohort studies of diabetes and bladder cancer risk based on rate ratio and hazard ratio. [file 1471-2407-13-310-S2.doc]

**Table 2 Characteristics of 19 cohort studies of diabetes and bladder cancer risk based on rate ratio and hazard ratio**

|  | **No. of**  **subjects** | **Demographics**  **of all subjects**  **(age, years; gender, %)** | **Diabetes**  **assessment** | **Bladder cancer assessment** | **Follow**  **up,**  **years** | **Adjusted RR (95% CI)** | **Adjustments** |
| --- | --- | --- | --- | --- | --- | --- | --- |
| Tripathi et al.  (USA) | 37,459 | Age: NA; f: 100% | Self-reported | Cancer registries | 13 | 2.46 (1.32-4.59)(f) | Age, smoking, physical activity, BMI, alcohol, occupation, marital status |
| Coughlin et al. (USA) | 1,056,243 | Age: 57; m: 44% | Self-reported | Death certificates | 16 | 1.43 (1.14-1.8)(m)  1.3 (0.85-2.0)(f)  1.4 (1.15-1.71)(both) | Age, race, years of education, BMI, smoking, alcohol, total red meat consumption, consumption of citrus fruits, juices and vegetables, physical activity, use of replacement estrogens |
| Jee et al. (Korea) | 1,298,385 | Age: 47; m: 64% | Self-reported or FGP | Cancer registry and hospitalization records | 10 | 1.32 (1.1, 1.57)(m) | Age, age squared, smoking, alcohol |
| Inoue et al. (Japan) | 97,771 | Age: 40-69; m: 48% | Self-reported | Cancer registries and hospitalization records | 10.7 | 1.63 (0.89-3.0) (m)  0.64 (0.09-4.75) (f)  1.5 (0.84-2.69)(both) | Age, study area, smoking, alcohol, BMI, physical activity, green vegetable and coffee intake, history of cerebrovascular or ischemic heart disease |
| Khan et al. (Japan) | 56,881 | Age: 40-79; m: 41% | Self-reported | Cancer registries and Death certificates | 8 | 1.03 (0.41-2.60)(m) | Age, BMI, smoking, alcohol |
| Larsson et al.  (Sweden) | 45,906 | Age: 45-79; m: 100% | Self-reported | Cancer registries | 9.3 | 1.16 (0.81–1.64)(m) | Age, education, smoking |
| Marianne et al. (USA) | 442,712 | Age: 54; m: 52% | Medical records or oral antidiabetic agent | Medical records | 3.5 | 2.35 (1.76-3.15) | Age, sex, schistosomiasis, pelvic radiation |
| Chung et al.  (Taiwan) | 54,751 | Age: 40-80; m: 100% | FGP and Self-reported | Death registry | 6.8 | 1.22 (0.27-5.61) | Age |
| Ogunleye et al. (UK) | 28,731 | Age: 62; m: 53.3% | NA | Cancer registries | 4 | 0.7 (0.4-1.21) | Deprivation decile |
| Tseng et al.  (Taiwan) | 998,947 | Age: NA; m: 49.6% | NA | NA | 3 | 1.49 (1.23-1.80) | Age, sex, living regions, occupation, systematic disease and oral drugs |
| Woolcott et al. (USA) | 185,816 | Age: 60.4; m: 48.1% | Self-reported | Cancer Registry and surveillance and national death index | 10.7 | 1.30 (1.07-1.57) | Smoking status, intensity and duration, and employment in a high risk industry |
| Lam et al.  (Asia, Australia) | 367,361 | Age: 48; m: 59% | Self-reported or blood glucose level | NA | 4.0 | 1.42 (0.70, 2.86) | Age |
| Seshasai et al.  (Europe, North America, Japan, other) | 820,900 | Age: 55; m: 52% | Self-reported, FPG, medication use, | Death certificates | 13.6 | 1.4 (1.01-1.96) | Age, smoking, BMI |
| Li et al. (USA) | 397,783 | Age: 46.8; m: 38.2% | Self-reported | Self-reported | NA | 1.7 (1.2-2.2)(m)  0.9 (0.6–1.3)(f)  1.33 (1.05-1.69)(both) | Age, race/ethnicity, health insurance coverage, smoking status, heavy drinking, BMI, physical inactivity |
| Wotton et al. (England) | 484,356 | Age: ≥30; m: 54% | Medical records | Death certificates | NA | 0.79 (0.64-0.97) | Age, sex, time period in single calendar years and district of residence |
| Atchison et al. (USA) | 4,501,578 | Age: 59.1; m: 100% | Hospital discharge diagnosis | Hospital admission  diagnosis | 11.7 | 0.96 ( 0.92-1.01) | Age, time, latency, race and number of visits, diagnoses of alcohol-related conditions, obesity and chronic obstructive pulmonary disease |
| Currie et al. (UK) | 112,408 | Age: 71.4; m: 48.1% | Read code indicative of diabetes | NA | 2.0 | 1.16 (1.02-1.32) | Age at baseline, sex, smoking, Charlson comorbidity index, year of diagnosis |
| Lee et al. (Taiwan) | 985,818 | Age: NA; m: 49.6% | Ambulatory and inpatient claims | Ambulatory and inpatient claims | 12 | 2.77 (2.37-3.22)(m)  2.53 (2.09-3.07)(f)  2.65 (2.35-2.98)(both) | NA |
| Liu et al. (Sweden) | 1,016,105 | Age: 67.0; m: NA | Hospital Discharge Register | Cancer Registry | 8 | 1.33 (1.18-1.49) | Age at diagnosis, sex, period, obesity, alcohol, smoking, socioeconomic status, and diagnosis region |

Abbreviations: *RR* relative risk, *CI* confidence interval, *DM* diabetes mellitus, *m* male, *f* female, *BMI* body mass index, *NA* data not available, FPG fasting plasma glucose
