# Supplementary material for: Risk of bladder cancer in patients with diabetes mellitus: an updated meta-analysis of 36 observational studies
Source: BMC Cancer. 2013 Jun 26;13:310. doi: 10.1186/1471-2407-13-310 (PMC3699355; doi:10.1186/1471-2407-13-310)
Supplement: Additional file 3: Table S3 — Characteristics of eight cohort studies of diabetes and bladder cancer based on standardized incidence/mortality ratio. [file 1471-2407-13-310-S3.doc]

**Table 3 Characteristics of eight cohort studies of diabetes and bladder cancer based on standardized incidence/mortality ratio**

|  | **No. of**  **subjects** | **Demographics**  **of all subjects**  **(age, years; gender, %)** | **Diabetes**  **assessment** | **Bladder cancer assessment** | **Follow**  **up,**  **years** | **Adjusted RR (95% CI)** |
| --- | --- | --- | --- | --- | --- | --- |
| Kessler et al. (USA) | NA | NA | NA | NA | NA | 0.71 (0.44-1.08) |
| Ragozzino et al. (USA) | 1,135 | NA | Medical records | Death certificates, autopsy reports, hospital records, outpatient visits | 8.6 | 1.49 (0.6-3.07) |
| Adami et al. (Sweden) | 51,008 | Age: NA; m: 45.4% | Discharge diagnosis | Cancer Registry | 5.2 | 1.0 (0.8-1.3)(m)  0.9 (0.6-1.3)(f)  0.98 (0.8-1.2)(both) |
| Wideroff et al. (Denmark) | 109,581 | Age: 66.5; m: 49.8% | Discharge records | Cancer registry | 5.7 | 1.0 (0.9-1.1)(m)  0.9 (0.8-1.1)(f)  1.0 (0.9-1.1)(both) |
| Verlato et al. (Italy) | 7,148 | Age: 66.6; m: 47.1% | Diabetes clinic, family physicians and drug prescription database | Death certificates | 10 | 1.33 (0.83–2.01)(m)  1.50 (0.55–3.26)(f)  1.36(0.91–1.98)(both) |
| Swerdlow et al. (UK) | 28,900 | Age: ＜50; m: 54.3% | National and geographical registers | Cancer Register | 18 | 1.0 ( 0.61 –1.55) |
| Tseng et al. (Taiwan) | 244,920 | Age: ≥25; m: 46.3% | NA | National Register of deaths | 12 | 2.93 (2.58-3.33)(m)  2.95 (2.49-3.5)(f)  2.94 (2.65-3.25)(both) |
| Hemminki et al. (Sweden) | 125,126 | Age: ＞39; m: NA | Hospital Discharge Register | Cancer Registry | 15 | 1.37 (1.25-1.49) |

*RR* relative risk, *CI* confidence interval, *m* male, *f* female, *NA*, data not available
