# Supplementary material for: Risk of bladder cancer in patients with diabetes mellitus: an updated meta-analysis of 36 observational studies
Source: BMC Cancer. 2013 Jun 26;13:310. doi: 10.1186/1471-2407-13-310 (PMC3699355; doi:10.1186/1471-2407-13-310)
Supplement: Additional file 4: Table S4 — Subgroup analysis of relative risks for the association between diabetes and bladder cancer risk. [file 1471-2407-13-310-S4.doc]

**Table 4 Subgroup analysis of relative risks for the association between diabetes and bladder cancer risk**

| **Subgroup** | **No. of studies** | **RR (95%CI)** | **Tests for heterogeneity** | | |
| --- | --- | --- | --- | --- | --- |
|  |  |  | Q | P | I^2^ (%) |
| Geographical region |  |  |  |  |  |
| Europe | 12 | 1.09 (0.96-1.24) | 53.05 | < 0.001 | 79.3 |
| North America | 13 | 1.36 (1.13-1.64) | 86.37 | < 0.001 | 86.1 |
| Asia | 9 | 1.84 (1.39-2.44) | 91.65 | < 0.001 | 91.3 |
| Other | 2 | 1.40 (1.04-1.89) | 0.00 | 0.971 | 0.0 |
| Publication year |  |  |  |  |  |
| 1970-1999 | 8 | 1.07 (0.93-1.23) | 14.48 | 0.043 | 51.7 |
| 2000-2005 | 7 | 1.52 (1.24-1.87) | 13.73 | 0.033 | 56.3 |
| 2006-2012 | 21 | 1.40 (1.13-1.72) | 600.95 | < 0.001 | 96.7 |
| Adjustment for age |  |  |  |  |  |
| Yes | 24 | 1.34 (1.18-1.51) | 140.02 | < 0.001 | 83.6 |
| No | 12 | 1.60 (1.53-1.67) | 354.94 | < 0.001 | 96.9 |
| Adjustment for sex |  |  |  |  |  |
| Yes | 9 | 1.36 (1.12-1.66) | 60.40 | < 0.001 | 86.8 |
| No | 27 | 1.34 (1.11-1.62) | 599.76 | < 0.001 | 95.7 |
| Adjustment for smoking |  |  |  |  |  |
| Yes | 18 | 1.32 (1.24-1.39) | 26.32 | 0.069 | 35.4 |
| No | 18 | 1.26 (0.99-1.60) | 630.84 | < 0.001 | 97.3 |
| Adjustment for alcohol use | | | | | |
| Yes | 9 | 1.32 (1.09-1.59) | 57.85 | < 0.001 | 86.2 |
| No | 27 | 1.35 (1.13-1.62) | 450.69 | < 0.001 | 94.2 |
| Adjustment for BMI |  |  |  |  |  |
| Yes | 11 | 1.35 (1.12-1.63) | 61.66 | < 0.001 | 83.8 |
| No | 25 | 1.34 (1.11-1.61) | 447.96 | < 0.001 | 94.6 |
| Adjustment for physical activity | | | | | |
| Yes | 5 | 1.43 (1.24-1.64) | 3.40 | 0.493 | 0.0 |
| No | 31 | 1.32 (1.13-1.55) | 653.75 | < 0.001 | 95.4 |

*RR* relative risk, *CI* confidence interval, *BMI* body mass index
